# A real-time imaging approach to quantify dendritic cell internalization for immunogenicity risk assessment of biotherapeutics

**DOI:** 10.3389/fimmu.2025.1632302

**Published:** 2025-09-12

**Authors:** Zhaojun Yin, Peter Tran, Joyce Guerrero, Justin Low, Qing Xie, Kun Peng

**Affiliations:** Department BioAnalytical Sciences, Genentech, Inc., South San Francisco, CA, United States

**Keywords:** anti-drug antibody, dendritic cell, immunogenicity prediction, internalization assay, real-time imaging

## Abstract

The presence of treatment-emergent anti-drug antibodies (ADAs) can pose challenges during biotherapeutic development by compromising drug safety and efficacy. Early assessment of immunogenicity risk is essential to mitigate these risks. Dendritic cells (DCs) are crucial for priming CD4 T cells and necessary for effective antibody production. Therefore, DC internalization has been investigated as a predictive tool for evaluating the immunogenicity risk of biotherapeutics. Previously reported flow cytometry-based DC internalization assays, including our own, have faced several limitations. These limitations included low throughput due to a restricted DC supply from healthy donors, restriction to Fc-containing antibody-based biotherapeutics, and offering only endpoint data. To address these limitations, we explored both direct and indirect labeling approaches using the IncuCyte® real-time imaging platform. Our findings revealed that indirect labeling approach with the commonly used Fab anti-Fc reagents was unsuitable for DC internalization assays due to significant assay background noises and assay bias when evaluating biotherapeutics of different frameworks. In contrast, using direct labeling with Biotracker Orange demonstrated improved predictability, required fewer DCs, and was suitable for high-throughput screening. Additionally, this approach facilitates constant monitoring of the internalization process, offering a comprehensive understanding of cell morphology changes and internalization kinetics. Using a panel of 25 test therapeutic antibodies with known clinical ADA results, the IncuCyte®-based direct labeling assay demonstrated significant improvements in predicting the immunogenicity risk of the tested molecules. The assay demonstrated a high correlation between internalization and clinical immunogenicity risk, outperforming our previous flow cytometry-based results. Overall, the IncuCyte®-based direct labeling assay using Biotracker Orange represents a significant advancement compared to our previous flow cytometry assay. This new technique is a valuable addition to our immunogenicity risk assessment toolbox, and will ultimately lead to more informed decision-making in the early development of biotherapeutics.

## 1 Introduction

Therapeutic proteins have revolutionized the treatment of a variety of severe and chronic conditions in the past two decades (1). Despite the advancement in protein engineering, immunogenicity still remains as one of the major challenges in the clinical development of therapeutic proteins, especially for immunomodulatory biotherapeutics and emerging modalities such as bispecific or multispecific antibodies (2–5). The undesirable immune responses in patients can lead to generation of anti-drug antibodies (ADAs), which can impact drug exposure, impair efficacy, cause serious safety concerns or even life-threatening events (6, 7). Therefore, immunogenicity risk assessment and management throughout the life cycle of biotherapeutic products is required by the health authorities. A strategy that integrates various aspects of the immunological mechanism underlying antibody responses is often used to evaluate the immunogenicity risk of the lead candidate in the early stages of drug development. The output of such an approach together with findings from preclinical studies and clinical factors (disease indication, dosing route and frequency etc.) could guide the bioanalytical strategy for immunogenicity monitoring, and the mitigation of potential efficacy and safety risks associated with immunogenicity during the clinical development of therapeutic proteins (8, 9).

The professional antigen presenting cells, mainly dendritic cells (DCs), are critical to initiate CD4 T cell activation that promotes generation of high affinity antibodies by B cells (10). The activation of naive CD4 T cells requires two signals: the interaction of T cell receptor (TCR) with peptide-MHC class II (pMHC-II) complex presented by DCs (signal 1), and the costimulatory signals provided by co-stimulatory receptors expressed on DCs (signal 2) (11). Immature DCs constitutively internalize and deliver the antigen to a multi-vesicular late endosomal-lysosomal antigen-processing compartment, known as the MHC class II compartment (MIIC). In MIIC, the antigen is degraded into short peptides and loaded onto MHC-II molecules (12, 13). Higher level of antigen internalization might contribute to increased pMHC-II complexes presented on the surface of DCs, which results in a more robust activation of CD4 T cells (14). Several studies have demonstrated that the level of internalization by DCs is positively correlated with the clinical ADA results of the test antibodies (15–17). The immature DCs must be properly activated or matured to elicit CD4 T cell responses by increasing the level of MHC-II and co-stimulatory receptors on the cell surface. Both patient conditions and characteristics of therapeutic proteins can contribute to the activation of DCs. For instance, there is evidence for increased DC maturation in rheumatoid arthritis (RA) patients, especially in the inflamed RA synovial tissues (18); protein aggregates have been demonstrated to cause remarkable DC activation in many studies (19–22). Several highly immunogenic therapeutic antibodies, such as bococizumab and briakinumab, have been shown to upregulate the expression of co-stimulatory receptors, i.e, CD86, CD83 or CD40, and chemokine receptor such as CXCR4 (23–25). However, the mechanism of DC activation by these antibodies, in the absence of large aggregates, remains poorly understood and requires further elucidation. Therefore, the DC internalization and activation assays are recognized as indispensable tools for understanding the immunogenicity risk of therapeutic proteins, which provide complementary insights to other *in vitro* assays such as MHC class II associated peptide proteomics (MAPPs) or T cell activation/proliferation assays (25–28).

Early drug development often involves the evaluation of multiple candidates, necessitating the use of high-throughput DC-based internalization assays to inform the selection of the lead candidate. Among many approaches developed to measure the internalization of therapeutic proteins in human DCs, most are flow cytometry based assays (15–17) that require a substantial number of cells and only work for Fc-containing biotherapeutics. This results in low assay throughput, consequently restricting the efficiency of rapid candidate screening across diverse modalities. While monocyte cell line THP-1 has been used as a substitute for DCs to enable higher throughput with comparable results as reported with a panel of therapeutic antibodies (29), concerns arise because monocyte cell lines, unlike primary DCs or monocyte-derived DCs used in our study, do not necessarily recapitulate the full functionality of APCs (10, 30). To address these limitations, herein we report the development of a real-time imaging-based high-throughput IncuCyte® internalization assay using DCs. This novel approach requires only 5-10% of the amount of DCs typically used in the traditional flow cytometry based assays and is capable of providing kinetic and cell morphology data in addition to the endpoint readout. Furthermore, the IncuCyte®-based DC internalization assay has demonstrated its capability to predict clinically immunogenic molecules using a panel of 25 therapeutic antibodies, offering a valuable tool to inform the selection of a lead candidate with low immunogenicity risk and thereby improve the probability of success in drug development.

## 2 Material and methods

### 2.1 Materials

The pharmaceutical grade-antibodies were obtained either internally (herceptin, bispecific mAb1) or purchased through Caligor Coghlan Pharma (aducanumab, bimekizumab, daratumumab, elotuzumab, eptinezumab, golimumab, lecanemab, nivolumab, pembrolizumab, sarilumab, satralizumab, tocilizumab, ustekinumab, vedolizumab). The research grade-antibodies, including ATR107, ATR107/Tras, bococizumab, briakinumab, canakinumab, dezamizumab, donanemab, dostarlimab, ebdarokimab, herceptin variants (**Table 1**), HuA33, ixekizumab, secukinumab, tildrakizumab, were generated in-house as described previously (4).

**Table 1:** Herceptin and 4 variants for evaluation.

Name	Isotype	Fc Modification
Herceptin_IgG1 (Herceptin)	IgG1	None
Herceptin_IgG2	IgG2	None
Herceptin_IgG4	IgG4	None
Herceptin_IgG1_N297G	IgG1	N297G
Herceptin_IgG1_LALAPG	IgG1	LALAPG

### 2.2 Antibody labeling and quality control

Antibodies were buffer exchanged into PBS pH 7.4 using Zeba 10K MWCO desalting columns (Thermo Scientific, Waltham, MA) according to the manufacturer’s protocol. After buffer exchange, antibody concentrations (in mg/mL) were determined using a spectrophotometer (A280 measurement divided by the 0.1% solution extinction coefficient) and diluted to 2 mg/mL with PBS pH 7.4. BioTracker Orange-NHS Live Cell pH Dye (Millipore, Burlington, MA) were then reconstituted to 10 mM by adding 107 µL of DMSO (Sigma-Aldrich, St. Louis, MO) to 1 vial (1 mg) of the dye, followed by vortexing at high speed for approximately 1 minute. A 5-fold molar excess of dye was added to each testing antibody in an amber tube, and conjugation reactions were allowed to proceed for 1 hour at room temperature on a rotator. After incubation, Zeba 40K MWCO desalting columns (Thermo Scientific) were used to remove free dye from the conjugates and formulate them in PBS pH 7.4 (per manufacturer instructions). Conjugate concentration and degree of labeling (DOL) were determined by measuring the absorbance of the conjugate at 280 nm and 551 nm and applying the following equations:


$$[protein]\;(mg/mL)\;=\frac{[A_{280}-\;(A_{551}\times \;CF_{280/551})]\;\times \;protein\;M_{w\;}(Da)\;\times \;DF}{\epsilon_{pr}}$$

$$DOL\;=\;\frac{A_{551}\;\times DF}{\epsilon_{551,\;pH\;7.4\;}\times [protein\;(M)]}$$

Where CF280/551 is the correction factor for absorbance of the BioTracker Orange at 280 nm at pH 7.4; DF is the dilution factor of sample being measured; ***ε***_pr_ is the molar extinction coefficient of antibody (M^-1^cm^-1^); ***ε*** 551, pH 7.4 is the molar extinction coefficient of the BioTracker Orange at 551 nm at pH 7.4 (M^-1^cm-1); protein (M) is the molar concentration of protein.

The distribution of BioTracer Orange conjugates is characterized by intact LC-MS as follows. The BioTracker Orange conjugates were deglycosylated with glycerol-free PNGase F and injected onto an Agilent PLRP-S (2.1 x 50mm, 8 μm particle size, 1000 Å pore size) column on a Sciex ExionLC™ UHPLC system coupled to X500B™ Q-TOF mass spectrometer. The separation gradient was employed from 25% to 90% mobile phase B (0.1% formic acid in 90% acetonitrile) over 6 min through mixing mobile phase A (0.1% formic acid in 2% acetonitrile) and B at a flow rate of 250 μL/min. The eluent was diverted into MS and the mass range for Q-TOF acquisition was set from 900 to 4400 m/z with accumulation time of 0.5 sec. The MS raw spectrum was deconvoluted and analyzed with BioTool kit within Explorer in Sciex OS software. The dye load distribution was determined by the ratio between peak area of each dye load subspecies to the total peak area of all species.

The aggregation level analysis of BioTracer Orange conjugates is performed by using Size Exclusion Chromatogaphy-Multi Angle Light Scattering (SEC-MALS), which was performed using a Thermo (Waltham, MA) Vanquish UHPLC with Waters (Milford, MA) SEC mAb column (200 Å, 2.5 µm, 7.8 x 150 mm) and 90:10 (200 mM K_3_PO_4_, 250 mM KCl, 0.02% (w/v) NaN_3_, pH 6.2):isopropanol (IPA) mobile phase at 0.5 mL/min flow rate. MALS was measured using a microDAWN light scattering detector and refractive index (RI) was measured using a microOptilab RI detector, both from Wyatt technology (Santa Barbara, CA). The molar mass of each species was determined using a refractive index increment (dn/dc) of 0.186 mL/g.

Example of SEC-MALS chromatograms and MS spectrum of BioTracker Orange conjugates were included in **Supplementary Figures S6**, **S7** respectively.

### 2.3 Generation of monocyte derived dendritic cell

DC generation and loading were conducted following a method published previously (16). In brief, monocytes were isolated using blood samples collected from anonymous healthy volunteers enrolled in the Genentech blood donor program, following a written informed consent approved by the Western Institutional Review Board. Monocytes were differentiated into immature DCs using interleukin-4 (R&D Systems, Minneapolis, MN) and granulocyte-macrophage colony-stimulating factor (GM-CSF; R&D Systems, Minneapolis, MN) for 5 days. The phenotype of immature DCs were characterized by staining cells with anti-CD11c (BioLegend, San Diego, CA), anti-CD209 (BioLegend, San Diego, CA) and anti-CD14 (BioLegend, San Diego, CA) on ice for 30 minutes. The cells were then washed twice with FACS buffer (2% FBS, 0.02% sodium azide in PBS), resuspended FACS buffer with 7AAD (Thermo Fisher Scientific, Eugene, OR), acquired on BD FACSCanto II, and analyzed using FlowJo version 10.10.0.

### 2.4 Internalization assay using indirect labeling with FabFluor orange or Zenon pHrodo green

Mix equal volume of 24 µg/mL FabFluor Orange (Sartorius, Ann Arbor, MI) or Zenon pHrodo Green (Thermo Fisher Scientific, Eugene, OR) and 24 µg/mL of each testing antibody prepared in DC medium (RPMI 1640, 10% FBS (Gibco, Grand Island, NY), plus 1% of the each following reagents: nonessential amino acids, sodium pyruvate, kanamycin sulfate (Gibco, Grand Island, NY)) and incubate at CO_2_ incubator (37 °C) for 30 minutes. DCs were washed twice with a pre-warmed (37 °C) DC medium and then counted using a Vi-CELL Blue cell counter (Beckman Coulter) to resuspend in the pre-warmed DC media to a final concentration of 0.4 × 10^6^ cells/mL. A 50 µL aliquot of the cell suspension was plated into each well of a 96-well clear flat-bottom tissue culture-treated microplate (Corning, Corning, NY), followed by the addition of 50 µL of 12 µg/mL conjugated testing antibodies. The microplate was then placed in the IncuCyte® SX5 (Sartorius, Ann Arbor, MI) for live cell imaging over 24 hours. The images were analyzed using IncuCyte® Software 2023A (Sartorius, Ann Arbor, MI) and the area of fluorescent signals was plotted with GraphPad Prism 10.4.1.

### 2.5 Internalization assay using direct labeling with BioTracker orange

DCs were washed twice with a pre-warmed (37 °C) DC medium. After washing, the cells were counted using a Vi-CELL Blue cell counter to resuspend in pre-warmed DC media to a final concentration of 0.2 × 10^6^ cells/mL. A 100 µL aliquot of the cell suspension was plated into each well of a 96-well clear flat-bottom tissue culture-treated microplate (Corning, Corning, NY), followed by the addition of 100 µL of 50 µg/mL conjugated antibodies. The microplate was then placed in the IncuCyte® SX5 (Sartorius) for live cell imaging over 24 hours. The images were analyzed using IncuCyte® Software 2023A (Sartorius) and the area of fluorescent signals was plotted with GraphPad Prism 10.4.1. Statistical analysis and plots were performed using JMP 18 or Scikit-learn package (1.2.1) and python (3.10.9). The overall charge of molecules was predicted using proprietary internal simulation tools.

For endpoint analysis, the sample signals were averaged over the final three hours. For slope analysis, a linear regression (GraphPad Prism 10.4.1) was performed using signals collected at six time points between the first and sixth hour.

The normalization was calculated as follows: (mean sample signal - mean herceptin IgG1 signal)/(mean bococizumab signal - mean herceptin IgG1 signal).

Due to the limited number of healthy donors (typically N=4) in the exploratory method development, formal statistical tests such as the t-test or ANOVA were not performed due to insufficient statistical power; instead, our analysis focused on the consistency of trends across individual biological replicates.

## 3 Results

### 3.1 Limitations of using an IncuCyte®-based indirect labeling DC internalization assay

**Figure 1:**
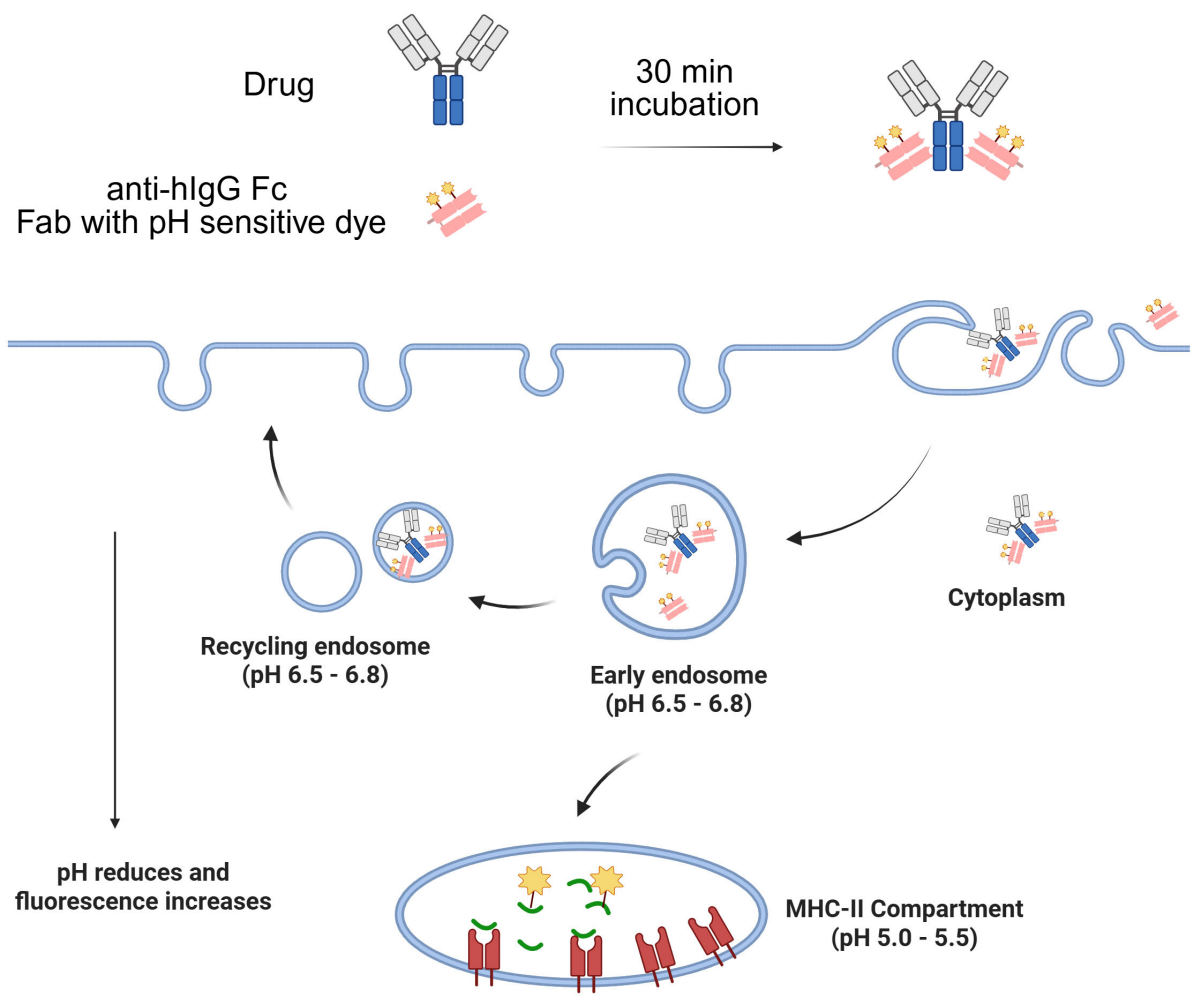
Illustration of Fab labeling approach to monitor Fc-containing antibody internalization by tracking the pH-sensitive dye in the acidic environments.

The most commonly utilized method for labeling Fc-containing testing antibodies involves briefly pre-incubating them with a Fab fragment of anti-Fc polyclonal antibodies conjugated with a pH-sensitive dye (31, 32). The resulting complex is then added to cells to monitor the testing antibody internalization by tracking the pH-sensitive dye in the acidic environments (**Figure 1**). This approach offers a quick and convenient way to track the test antibodies without the need for direct conjugation with dyes.

#### 3.1.1 Using FabFluor indirect labeling led to high uptake of free reagent and large assay variabilities among IgG isotypes and Fc mutations

**Figure 2:**
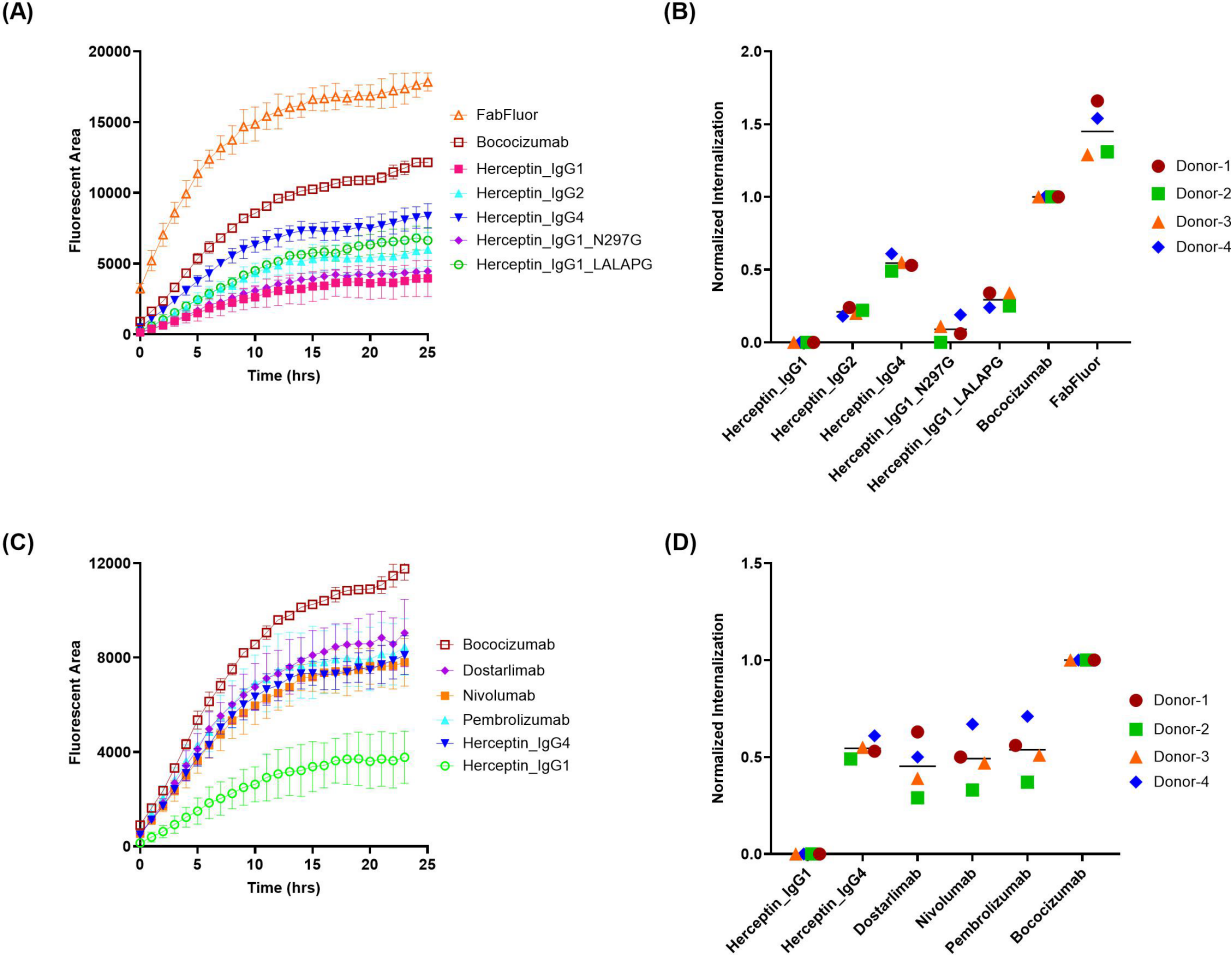
FabFluor-pH Orange indirect labeling assay shows distinct internalization profiles of antibody isotypes and Fc variants. The internalization of herceptin variants and IgG4 molecules into DCs were evaluated using IncuCyte^®^ by indirect labeling with FabFluor-pH Orange. **(A, C)** representative time-course internalization of **(A)** five herceptin variants and **(C)** four IgG4 molecules from a single donor. Data points are averages of duplicate wells. **(B, D)** Endpoint internalization after 24 hours for **(B)** five herceptin variants and **(D)** four IgG4 molecules. Data were acquired from four independent donors across multiple days, normalized as described in the Materials and Methods.

We first evaluated a commercial reagent, IncuCyte® FabFluor orange, which is a proprietary pH-sensitive dye provided by Satarious. A panel of 5 herceptin variants (**Table 1**), including herceptin of the wild type IgG1, IgG2, IgG4 isotypes and two Fc effector function attenuated mutants (IgG1 [N297G] and IgG1 [LALAPG]), were generated to assess the impact of IgG isotypes and Fc on MoDC internalization by comparing with benchmark molecules with known low (herceptin, IgG1) and high (bococizumab, IgG2) level of internalizations respectively (4). Additionally, the FabFluor orange without any testing antibodies was included as a control to determine the internalization of the free labeling reagent. As shown in the time-course internalization results (**Figure 2A**), the internalization of bococizumab is substantially higher than herceptin over time with a signal approximately 3 times that of herceptin after 24 hours, clearly distinguishing between the positive and negative controls for practical application. However, there was a wide range of internalization signals among the 5 herceptin variants. For example, the internalization signal for herceptin IgG4 after 24 hrs is nearly twice as that of IgG1. The average normalized DC internalization of the 5 herceptin variants from four healthy donors indicates a trend of assay signals with herceptin IgG4 > herceptin IgG1 LALAPG > herceptin IgG2 > herceptin IgG1 N297G ≈ herceptin IgG1 (**Figure 2B**). Notably, the free Fabfluor reagent showed the most internalization in DCs, indicating interference from unbound Fabfluor, which could lead to false positive signals. To determine whether the observed high internalization of IgG4 isotype is exclusive to herceptin IgG4, three commercial anti-PD1 IgG4 mAbs (dostarlimab, nivolumab, pembrolizumab) were evaluated, which are anticipated to have low internalization due to the lack of PD-1 expression on DCs and their low clinical ADA incidences (2.5% for dostarlimab, 9% for nivolumab, and 2.1% for pembrolizumab) (33). Similar to herceptin IgG4, the internalization of three anti-PD1 IgG4 mAbs also had signals much higher than that of herceptin IgG1 (**Figures 2C, D**). Therefore, the FabFluor indirect labelling approach does not allow an unbiased comparison of the DC internalization of antibody candidates with different isotypes and/or Fc mutations.

#### 3.1.2 Zenon pHrodo green indirect labeling resulted in reduced free reagent uptake and variabilities among IgG isotypes and Fc mutations

**Figure 3:**
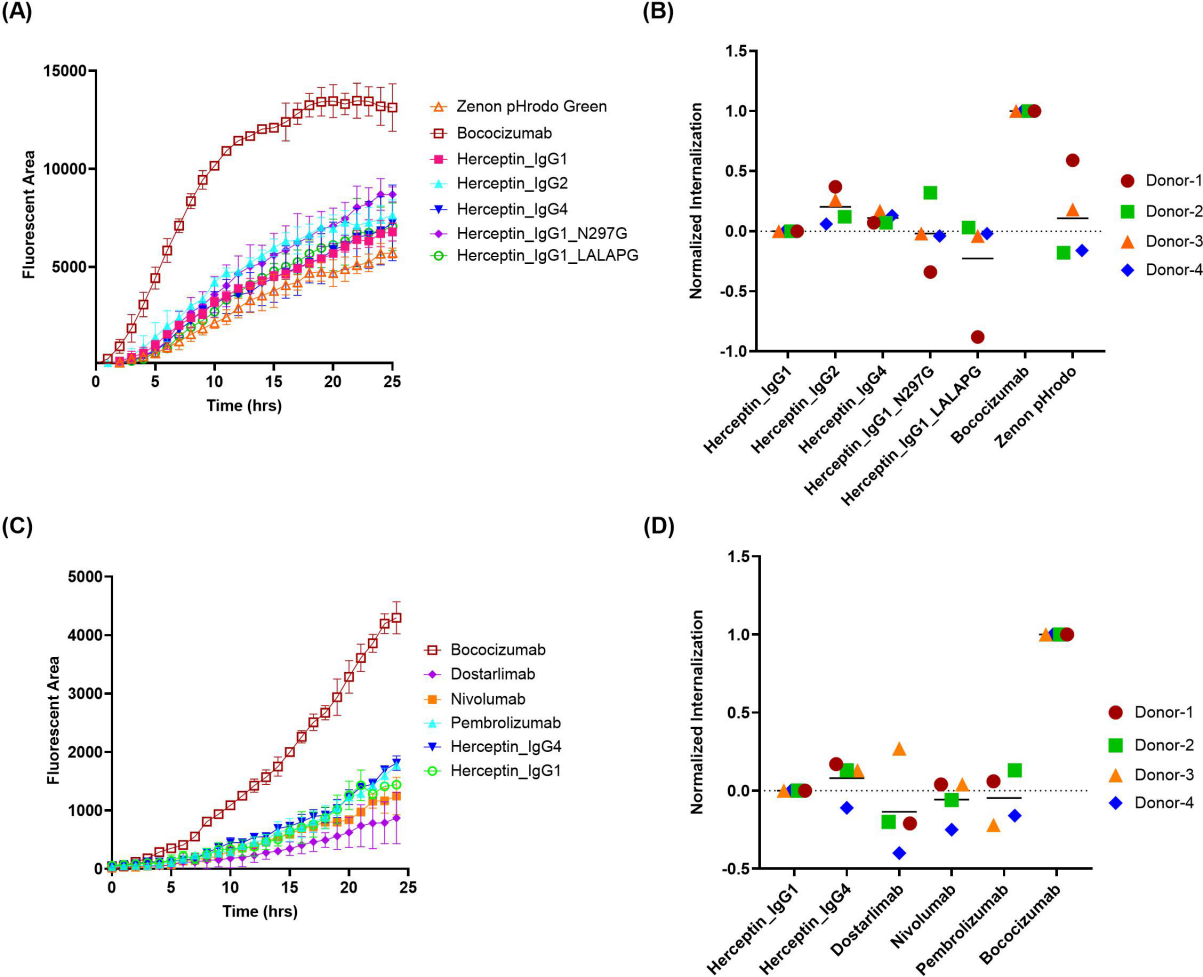
Quantification of antibody internalization into DCs using a Zenon pHrodo Green indirect labeling assay. The internalization of herceptin variants and IgG4 molecules into DCs were evaluated using IncuCyte^®^ by indirect labeling with Zenon pHrodo Green. **(A, C)** representative time-course internalization of **(A)** five herceptin variants and **(C)** four IgG4 molecules from a single donor. Data points are averages of duplicate wells. **(B, D)** Endpoint internalization after 24 hours for **(B)** five herceptin variants and **(D)** four IgG4 molecules. Data were acquired from four independent donors across multiple days, normalized as described previously.

Next, a commercial reagent Zenon pHrodo green was evaluated, which is another pH-sensitive dye conjugated to Fab fragment of anti-Fc polyclonal antibodies. In contrast to FabFluor orange, the uptake of free Zenon pHrodo green is relatively low, similar to the low ADA control, herceptin IgG1 (**Figures 3A, B**). Furthermore, the variability among the 5 herceptin variants is lower compared to FabFluor orange, and even the internalization of herceptin IgG4 is not significantly different from herceptin IgG1. The three commercial anti-PD1 IgG4 mAbs all exhibited relatively low internalization signals comparable with the negative control (**Figures 3C, D**).

Although Zenon pHrodo green demonstrated improved performance compared to FabFluor, there are remaining challenges and issues. First, highly immunogenic mAbs, such as briakinumab (ADA: 70%) and ATR-107 (ADA: 76%), are poorly internalized in DCs when using Zenon pHrodo green (**Supplementary Figure S1** in **Supplementary File**). Moreover, we have observed that the performance of Zenon pHrodo green internalization tends to be influenced by the differentiated state of DCs. The poorly differentiated DCs, characterized by high CD14 and low CD209 expression, typically result in high internalization of herceptin and minimal separation between herceptin and bococizumab (**Supplementary Figure S2** in **Supplementary File**). Those aspects need to be elucidated further to fully understand and mitigate these limitations.

### 3.2 Development of an IncuCyte®-based DC internalization assay using direct labeling with BioTracker orange

The challenges encountered in the indirect label work prompted exploring direct conjugation of therapeutic proteins with a pH-sensitive dye as an alternative approach. While several pH-sensitive dyes such as pHrodo have been widely used in literature, we found that BioTracker Orange, also known as AcidiFluor Orange, offers several advantages over other commercial alternatives. Notably, BioTracker Orange exhibits a remarkable 50-fold increase in fluorescence when transitioning from a neutral pH of 7.4 to an acidic pH of 5.0, providing a highly sensitive measurement of internalization events within the MHC-II compartment (34). Additionally, it demonstrates excellent resistance to photobleaching, enabling repetitive imaging and long-term kinetic studies. Moreover, proteins labeled with Biotracker Orange typically maintain good stability, with preserved molecule integrity and biophysical properties. These attributes make Biotracker Orange an ideal selection for developing internalization assays using the IncuCyte® platform.

**Figure 4:**
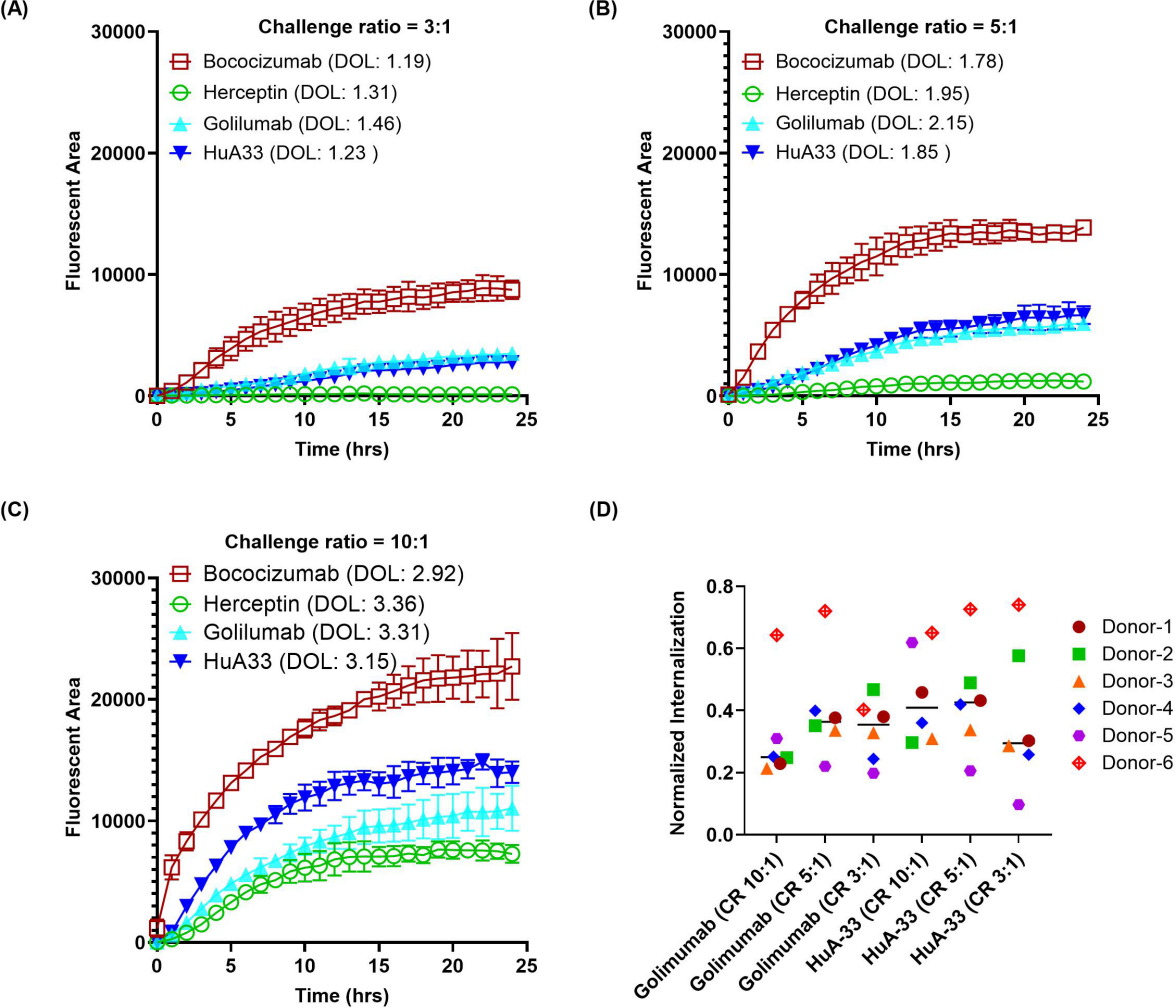
Effect of dye-to-protein challenge ratio on antibody internalization. Antibodies were directly labeled with Biotracker Orange dye at various challenge ratios (CR) to assess the impact of the resulting degree of labeling (DOL) on internalization by DCs. **(A–C)** Representative time-course internalization profiles for antibodies labeled at a CR of **(A)** 3:1, **(B)** 5:1, and **(C)** 10:1. Data were obtained from a single donor, and points represent the average of duplicate wells. **(D)** Endpoint internalization after 24 hours, comparing the different labeling conditions. Data were acquired from six independent donors across multiple days, normalized as described previously.

Optimization of the dye-to-protein labeling ratio is one of the most critical parameters for the direct conjugation approach, as a high dye-to-antibody labeling ratio might increase antibody instability, while a low labeling ratio might reduce assay sensitivity. Additionally, the labeling of proteins might alter their biophysical properties (35), which is a significant factor in their internalization by DCs. To identify the optimal labeling ratio of BioTracker Orange, we evaluated a range of dye-to-antibody challenge ratios from 3:1, 5:1, to 10:1, using one highly immunogenic molecule HuA33 (ADA: 73%) and one intermediate immunogenic molecule golimumab (ADA: 21%) in addition to the two benchmark controls herceptin and bococizumab. The actual degree of labeling (DOL) measured by absorbance ranged from 1.3 to 3, corresponding to the challenge ratios of 3:1 to 10:1. The intact LC-MS analysis (**Supplementary Table S1** in **Supplementary File**) indicated that the residual unconjugated mAb was minimal at the 10:1 challenge ratio (11 ~ 17%) but was more pronounced at the 5:1 ratio (31 ~ 41%) and even higher at the 3:1 ratio (48 ~ 56%). However, the proportion of heavily labeled species, i.e., five dyes and above per mAb, significantly increased when challenge ratio was increased from 3:1 (0%) to 5:1 (0 ~ 2.4%) and further to 10:1 (10 ~ 23%). Increasing the number of dyes per mAb might elevate the risk of altering the biophysical properties of the mAb, potentially impacting its interaction with DCs. As illustrated in **Figures 4A-C**, using antibodies conjugated at higher challenge ratios resulted in stronger signals, but it could lead to reduced separation between the intermediate molecules and the low immunogenic molecule, as observed with golimumab. **Figure 4D** shows that a normalization approach using positive and negative controls suggests the 5:1 challenge ratio yields the best normalized internalization for both golimumab and HuA33. It’s important to acknowledge the notable inter-donor variability in these data (e.g., donor #6), which is expected due to the biological heterogeneity of primary human monocyte-derived dendritic cells. This observation underscores the necessity of using an appropriate number of donors in routine evaluations to ensure conclusions are robust and account for such biological variance. Based on those observations, a 5:1 challenge ratio was selected to provide adequate resolution between intermediate/highly immunogenic molecules and the low immunogenic molecules. This challenge ratio also offers sufficient buffer room for conjugation while minimally perturbing the biophysical properties of the proteins.

To confirm the optimal labeling ratio, a panel of monospecific antibodies (mAbs) and bispecific antibodies (bsAbs) labeled with a 5:1 (dye: antibody) challenge ratio were evaluated, using DCs derived from three healthy donors. The 5 tested mAbs with known clinical ADA incidence included bococizumab (ADA: 48%), ATR-107 (ADA: 76%), briakinumab (ADA: 70%), golimumab (ADA: 21%), and herceptin (ADA: 0.1%). The 2 tested bsAbs included an internal candidate bsAb1 (ADA: 8.5%) and a hybrid bsAb (ATR-107/Tras, no ADA data) with the two variable domains from the highly immunogenic ATR-107 and low immunogenic herceptin (4).

**Figure 5:**
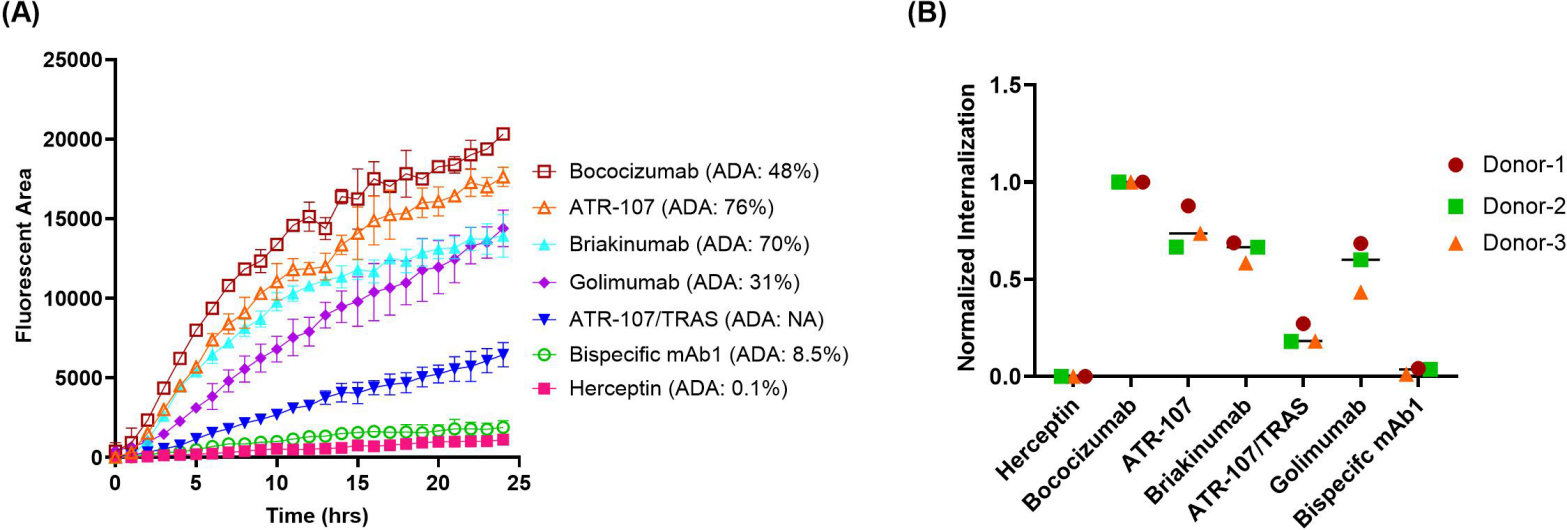
Quantification of antibody internalization into DCs using direct labeling with Biotracker Orange. The internalization of five monoclonal antibodies and two bispecific antibodies, directly labeled with Biotracker Orange, was quantified in DCs using IncuCyte^®^. **(A)** Representative time-course of internalization for all tested antibodies using cells from a single donor. Data points represent the average of duplicate wells. **(B)** Endpoint analysis after 24 hours. Data were acquired from three independent healthy donors and normalized as described previously.

As shown in **Figure 5**, the highly immunogenic antibodies in the clinic, such as bococizumab, ATR-107, and briakinumab, exhibited significantly higher internalization compared to the negative control herceptin. This trend was also observed with golimumab, which has an intermediate ADA incidence, although to a lesser extent. The bsAb ATR-107/Tras demonstrated intermediate internalization that was significantly different from both ATR-107 and herceptin mAbs. Our internal bsAb1 with a low clinical ADA rate had minimum internalization similar to that of herceptin.

### 3.3 Panel test using Incucyte-based direct labeling method

**Figure 6:**
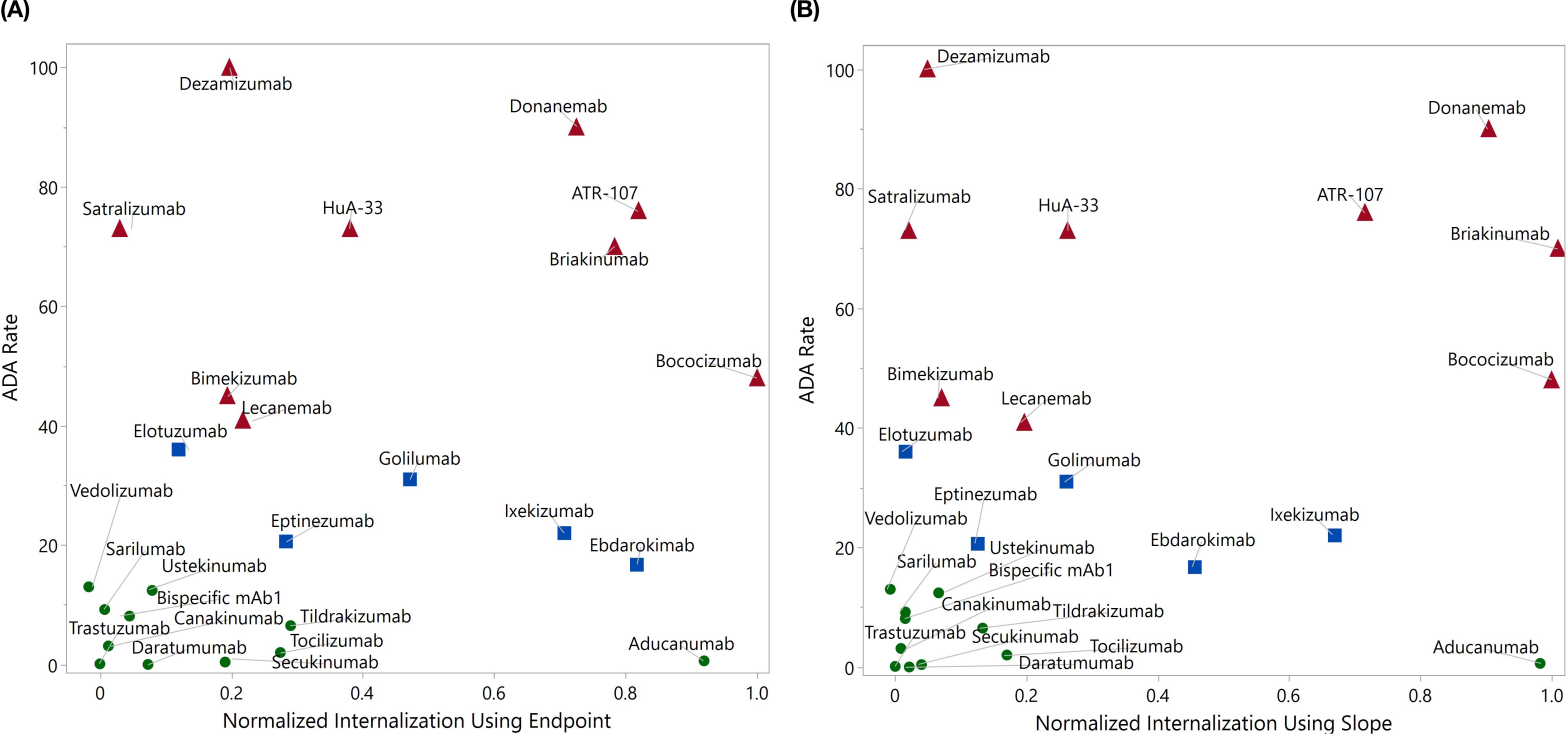
Panel test using direct labeling with BioTracker Orange. The average normalized internalization index for each molecule using endpoint (**A**, up to 24 hrs) and slope (**B**, up to 6 hrs) was plotted against the clinical ADA incidence rate. The drugs with ADA greater than 40% were marked in red, those with ADA between 15% and 40% were marked in blue, and the remaining drugs were marked in green.

After establishing the optimal assay protocol, we expanded our investigation to assess a larger panel of mAbs with documented clinical ADA results. These 21 mAbs were selected based on a couple of considerations. First of all, the selected antibodies exhibit a broad range of clinical ADA incidences, spanning from nearly zero to 100%; second, many of the selected antibodies with intermediate clinical ADAs demonstrated minimal internalization in our previously developed flow based DC internalization assay (16). Additionally, the results from four test molecules used in early assessments were integrated, bringing a total of 25 test molecules. The average normalized DC internalization using endpoint (**Figure 6A**) or slope as the kinetic readouts (**Figure 6B**) was plotted against the corresponding clinical ADA rate of the testing mAbs. We incorporated slope analysis to address the potential limitation of relying solely on endpoint analysis. The time to reach a response plateau can vary considerably depending on the molecule’s properties, target expression, and donor variability. This kinetics variability means that the choice of specific time points could lead to contradictory results. For instance, the internalization curves for ebdarokimab and donanemab intersect at approximately 15 hours (**Supplementary Figure S3** in **Supplementary File**). Depending on the selected time point, one could conclude either donanemab (before 15 hours) or ebdarokimab (after 15 hours) has a higher DC internalization.

**Figure 7:**
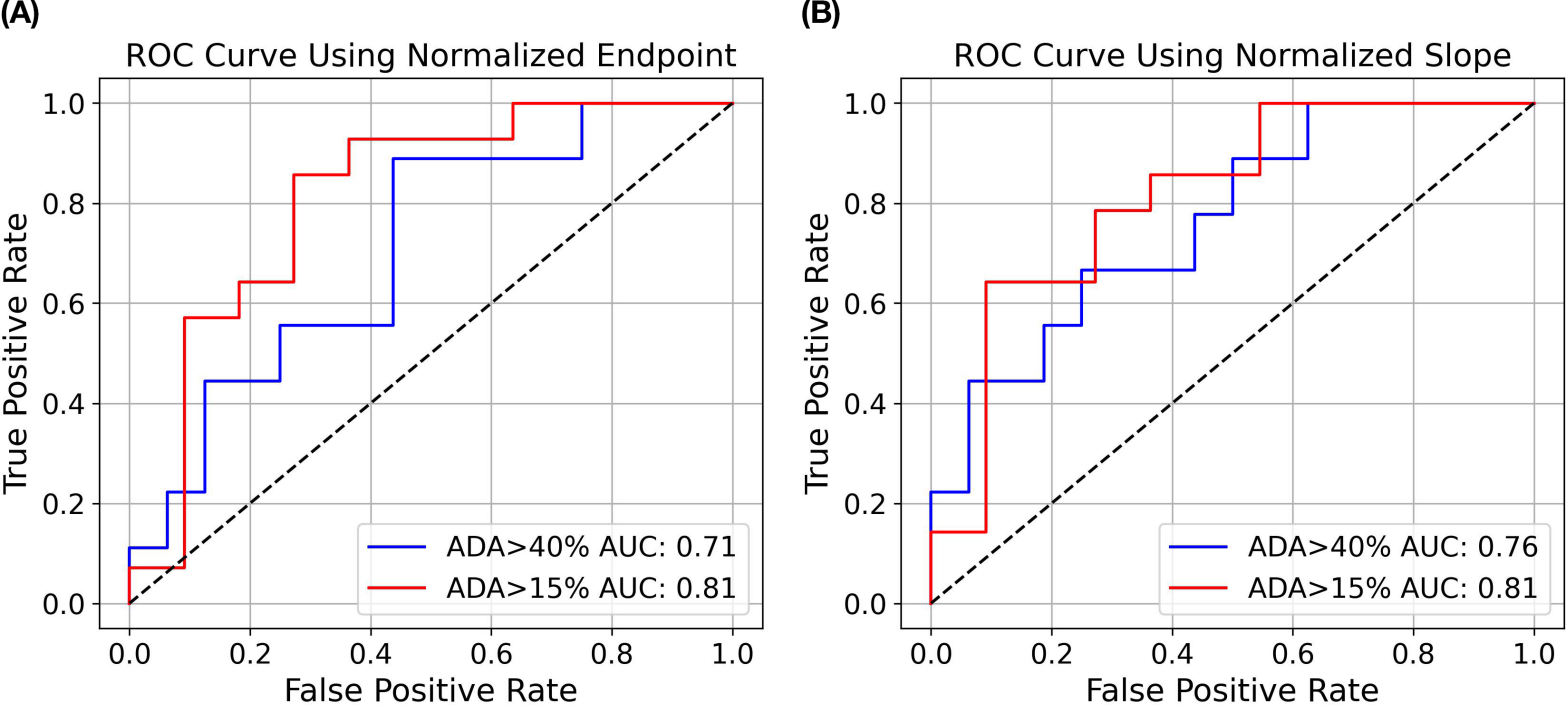
Logistic regression modeling to predict ADA status from DC internalization. Receiver Operating Characteristic (ROC) curves show the performance of logistic regression models using endpoint **(A)** and slope **(B)** analysis as described previously. The blue curve illustrates the AUC for ADA>40% versus ADA<40%, while the red curve illustrates the AUC for ADA>15% versus ADA<15%.

Compared to the endpoint analysis, the slope analysis is much more compact on the distribution of drugs with ADA < 15% (excluding aducanumab), which may facilitate better distinction between drugs with high and low ADAs. As demonstrated in the logistic regression analysis in **Figure 7**, the area under the ROC curve (AUC) for slope analysis is greater than that for endpoint analysis (0.76 vs 0.71, p-value: 0.056 vs 0.112) in distinguishing ADA>40% and ADA<40%. Nevertheless, both analyses exhibit strong distinction between drugs with ADA > 15% and ADA < 15% (AUC = 0.81 for both, p-value: 0.037 vs 0.018)). This underscores the assay’s efficacy in distinguishing molecules with intermediate or high immunogenicity from those with low immunogenicity. In contrast, our previous flow cytometry-based internalization has a poor performance with endpoint analysis (15% ADA cutoff: AUC = 0.60, p = 0.163; 40% ADA cutoff: AUC = 0.53, p = 0.862) (**Supplementary Figure S4** in **Supplementary File**). It is important to note that the model’s findings are preliminary and derived from a limited size of testing molecules. To validate and enhance the reliability of the model, it is crucial to further evaluate a much larger panel of molecules with diverse targets and biophysical properties. This will help to provide a comprehensive assessment of the assay’s ability to differentiate immunogenic molecules and eventually establish a robust predictive internalization threshold for routine screening.

In addition to kinetics data, the direct label IncuCyte® assay also collects valuable morphological information, such as real-time changes in DC morphology (**Supplementary Figure S5** in **Supplementary File**), which can inform additional immunogenicity studies. Ongoing research focuses on leveraging these morphology data to better understand the mechanisms of biotherapeutic internalization and immune activation.

## 4 Discussion

DCs play a pivotal role in priming CD4 T cells, which are essential for effective antibody production. Unlike B cells, macrophages, or monocytes, DCs are professional antigen-presenting cells with a unique ability to capture, process and present antigens to naive CD4 T cells. Upon capturing antigens, DCs migrate to lymphoid tissues where they present the processed antigens via MHC class II molecules. This interaction, along with co-stimulatory signals provided by DCs, is vital for the activation and differentiation of CD4 T cells into helper T cells, which consequently provide necessary signals to B cells to initiate and enhance antibody production. For those reasons, DC internalization (15–17), activation through costimulatory signals (24, 25), and migration mediated by chemokine receptors (23) have been investigated as predictive tools to evaluate the immunogenicity risk of biotherapeutics.

The internalization and processing of antigens by DCs is a sophisticated and tightly regulated process. Once an antigen is encountered, DCs internalize it through mechanisms such as phagocytosis, receptor-mediated endocytosis, or macropinocytosis. In addition, for Fc containing therapeutics, the net intracellular accumulation within DCs is not solely governed by the rate of uptake but is also critically modulated by intracellular recycling dynamics. Extensive evidence shows that neonatal Fc receptor (FcRn) rescues IgG from lysosomal degradation by binding it within the acidic endosome and recycling it back to the cell surface, thereby influencing antibody accumulation and potential immunogenicity (36–41).

Our previously developed flow cytometry-based DC internalization assay demonstrated a positive correlation between the level of internalization and clinical immunogenicity risk in a panel of 8 testing antibodies. However, several limitations were identified, especially when exploring more diverse modalities. It struggles with molecules that have high levels of target expression on the DC surface, such as PD-L1, due to the extensive surface staining that obscure internalization signals. Additionally, the assay often fails to capture the initial rapid receptor-mediated endocytosis and has poor sensitivity in detecting molecules with intermediate levels of ADAs. Furthermore, the assay is time-consuming and requires a substantial number of DCs per sample analysis (around 10^6^ cells/sample), posing challenges to use it in high-throughput screening during the early drug discovery phase. Those challenges highlight the need for alternative methods to accurately assess the immunogenicity risk of various biotherapeutics.

A method using (Fab′)_2_ anti-Fc antibodies with a pair of FRET dyes to label the test mAbs has been previously reported, which activates fluorescence when the complex is digested in the lysosome (17). Recently, a method incorporating a pH-sensitive dye into the N-glycan of antibody has been reported, which leverages the pH differential between the extracellular environment and the acidic MHC-II compartment (15). To address the limitations identified in our previous flow cytometry-based DC internalization assay, we are particularly interested in the pH-sensitive dye approach due to its straightforward implementation. We started investigating the IncuCyte® real-time imaging platform, which requires significantly fewer DC cells (4 x 10^4^ cells/sample for duplicates), making it a more attractive solution for high-throughput screening. Furthermore, the IncuCyte® platform offers real-time analysis to provide continuous monitoring of cell morphology and internalization processes, leading to a more comprehensive understanding of the kinetics involved.

Fab anti-Fc antibodies labeled with pH-sensitive dyes have been widely used in studying the receptor-mediated internalization of antibodies using the IncuCyte® platform (42–44). These reagents are commercially available and can be applied to any Fc-containing molecules, providing a convenient and user-friendly method without the need for directly modifying the test molecules. This method has proven to be effective in assessing target mediated antibody internalizations. However, the majority of the tested biotherapeutics, even the highly immunogenic ones (e.g. bococizumab or briakinumab), do not have specific targets on DCs, suggesting that their uptake by DCs may primarily occur through macropinocytosis. The non-receptor mediated uptake by these biotherapeutics on IncuCyte® platform using Fab anti-Fc antibodies labeled with pH-sensitive dyes has not been thoroughly investigated.

During assay development of the indirect labeling approach, we explored multiple pH-sensitive dyes conjugated to Fab anti-Fc polyclonal antibodies. Both FabFluor Orange and Zenon pHrodo dyes were capable of differentiating the benchmark molecules of high and low immunogenicity. However, free FabFluor Orange exhibited very high levels of internalization and led to high assay background and poor assay sensitivity. Additionally, method biases were observed when testing antibodies with Fc modifications and various IgG isotypes using FabFluor Orange. This is most likely due to different binding affinities between the Fab reagent and various IgG frameworks, resulting in varying levels of free dye that produce inconsistent signals. Given the limited dynamic range between high and low immunogenic benchmark molecules, these variabilities caused by frameworks are significant and cannot be overlooked. Minimizing the impact of frameworks by normalizing with a framework-matching control is a potential solution, however, it would be extremely resource and time-consuming, especially considering the diverse IgG isotypes and Fc mutations applied in modern antibody engineering (45, 46). This outcome highlights a unique challenge for DC internalization assays that is different from the traditional antibody candidate screening by monitoring the receptor-mediated internalization, which is a usually much more robust process than macropinocytosis with candidate clones typically generated on the same framework. However, immunogenicity assessments often involve comparing molecules with different frameworks and/or Fc modifications, and the internalization signals are not as robust, which can introduce variability and complicate the analysis. While Zenon pHrodo green has significantly improved overall assay performance over FabFluor Orange, several limitations remain. The method requires high quality of fully differentiated dendritic cells, and the internalization of highly immunogenic benchmark molecules such as briakinumab and ATR-107 remains very low for unknown reasons. The immune complex formed between Fab anti-Fc polyclonal antibodies and the test mAbs might differ in size, which could potentially affect the internalization of specific molecules. Therefore, while Zenon pHrodo green represents a step forward, additional refinement and investigation are required to optimize the internalization assay for the evaluation of biotherapeutics with diverse characteristics.

To circumvent the limitations of indirect labeling, we implemented a direct labeling strategy applicable to a wide range of biotherapeutics beyond Fc-containing molecules. Although the labeling and purification process involves additional steps, it also allows an improved quality control of the conjugates to provide cleaner and more accurate results, without the concern of the high assay background introduced by non-specific uptake of free reagents and assay biases caused by framework differences. We chose pH-sensitive dyes for this strategy due to their versatility and potential for future multiplexed analysis. Biotracker Orange was selected due to its superior sensitivity to pH changes, resistance to photobeaching and good stability.

DOL is a critical parameter in the direct labeling approach. Our strategy is to use the lowest DOL possible without compromising assay sensitivity, thereby minimizing any modification on the properties of the molecules. The optimization indicated a challenge ratio of 5:1 that yields a typical DOL of approximately 2.0. In practice, the DOL for any test molecule is typically maintained between 85% and 115% of the DOL of negative control benchmark (herceptin) to prevent false negative and positive. This ensure the assay remain sensitive and specific while minimizing alternations to the properties of the test molecules.

To further evaluate the performance of IncuCyte®-based assay, a total of 25 mAbs were analyzed using either endpoint analysis or slope analysis. With endpoint analysis up to 24 hrs, the IncuCyte®-based assay demonstrated superior performance compared with previously developed flow cytometry based internalization assay in discriminating molecules with ADA cutoff of either 40% or 15%. This improvement is mainly due to the increased signals in low pH antigen processing compartments and better sensitivity provided by the accumulation of labeled molecules over time.

One limitation of endpoint analysis is that it does not reveal whether the internalization signal has reached a plateau or not. The kinetics data by IncuCyte®-based assay could provide insights whether the endpoint analysis at a specific time point is appropriate. Therefore, we also performed slope analysis up to 6 hours, during which highly internalized molecules have not yet reached their plateau. Slope analysis is valuable because it tracks the rate of internalization over time, rather than just the final amount internalized at a single time point. This can provide additional insights into the dynamics of molecule internalization, revealing differences between molecules that may reach similar endpoint values but differ in their initial internalization rates, or achieve high endpoint values but with slow internalization rates. As shown in **Figures 6** and **7**, slope analysis offers better separation with ADA cutoff of 40%, which offers more confidence in distinguishing highly immunogenic molecules from those with low or intermediate immunogenicity. Conversely, endpoint analysis might be more suitable to differentiate low and intermediate ADA molecules, as the latter needs more time to accumulate the signal. Future analyses with more test molecules are warranted to further validate these findings.

It should also be noted that several mAbs exhibited internalization levels that did not correlate with clinical ADA rates. Aducanumab, which reduces Amyloid-beta for treating Alzheimer’s disease, showed a similar high level of internalization as bococizumab, although its clinical ADA rate is only 0.6%. One possible explanation is that aducanumab has an extremely high Fv (variable region) charge, approximately 11.3, which is even slightly higher than that of bococizumab (10.5) (47). A highly positive Fv charge can cause non-specific binding due to the increased interaction with negatively charged cell membranes components, such as phospholipids and glycosaminoglycans. Conversely, satralizumab, an anti-IL6R molecule, exhibited extremely low internalization (normalized endpoint internalization index = 0.03), despite having a clinical ADA rate of 73%. Interestingly, tocilizumab, which shares the same mechanism of action (MOA) as satralizumab, showed moderate internalization (0.27) while its clinical ADA rate is only about 2%. There are two factors that might contribute to the low internalization of satralizumab. First, the antibody recycling technique was incorporated in designing satralizumab, which allows it to dissociate from IL6R in pH dependent manner, helps prevent the receptor mediated degradation of the molecules (48). Second, satralizumab has a slightly negative Fv charge of -1.3, which could reduce the nonspecific binding, whereas tocilizumab has a very high positive Fv charge of 9.0. However, this discrepancy in ADA rate could not be fully explained and warrants further investigation, especially the presence and nature of T-cell epitopes and their potential to activate CD4 T cells. This observation underscores the importance of considering molecule specific characteristics to inform DC data interpretation.

While the IncuCyte®-based DC internalization assay demonstrated a good correlation with clinical ADAs in the tested molecules, the internalization assay alone should not be used exclusively for immunogenicity risk assessment. Immunogenicity is a complex phenomenon that often requires integrating multiple techniques and information, such as Mass Spectrometry-Based Peptides Presentation (MAPPs), T-cell activation and proliferation assays, the mechanism of action, and even clinical factors like patient demographics, disease state, and treatment regimen. Combining these methods can provide a more comprehensive understanding of immunogenicity risks, ultimately supporting better-informed decision-making in the development of biotherapeutics.

## 5 Conclusion

An IncuCyte®-based direct labeling DC internalization assay was successfully developed as a predictive tool for immunogenicity risk assessment. This assay demonstrates significantly improved predictability compared to the previous flow-based assay, with a reduced number of DCs required and the experimental setup is operational friendly. It should be emphasized that the pH sensitive dye labeling, whether directly or indirectly, has the potential to alter the physicochemical properties of test molecules by introducing additional charges or increasing aggregation. Therefore, the internalization of biotherapeutics should be evaluated on a case-by-case basis.

In summary, the IncuCyte®-based direct labeling DC internalization assay represents a significant advancement in immunogenicity assessment compared to the previous flow cytometry assay, and is a valuable addition to in immunogenicity prediction toolbox. It provides more informative and sensitive data while facilitating a more comprehensive analysis, which enables better decision-making in the evaluation and development of biotherapeutics, when combined with additional methodologies and ongoing research.

## Data Availability

The original contributions presented in the study are included in the article/**Supplementary Material**, further inquiries can be directed to the corresponding author.
