# Supplementary material for: A real-time imaging approach to quantify dendritic cell internalization for immunogenicity risk assessment of biotherapeutics
Source: Front Immunol. 2025 Sep 12;16:1632302. doi: 10.3389/fimmu.2025.1632302 (PMC12463596; doi:10.3389/fimmu.2025.1632302)
Supplement: Supplementary file 1 [file Presentation1.zip › Suppl. Images.docx]

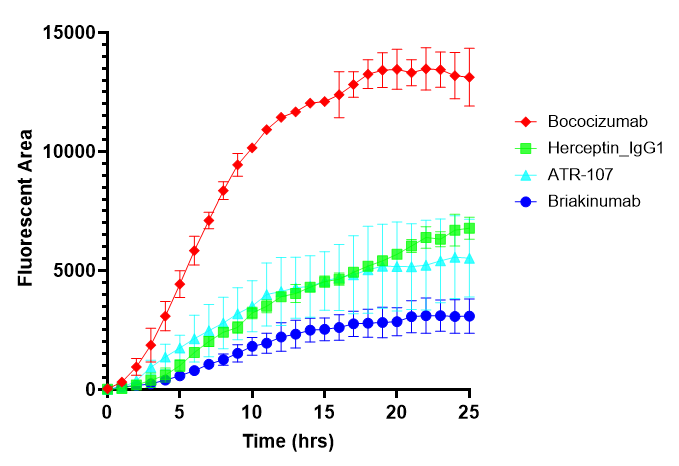


**Figure S1**. Both high ADA benchmarks, briakinumab and ATR-107, failed to demonstrate high internalization with Zenon pHrodo green labeling, while both mAbs constantly showed high internalization in our previous flow cytometry based assay or incuCyte assay using direct labeling with Biotracker Orange.


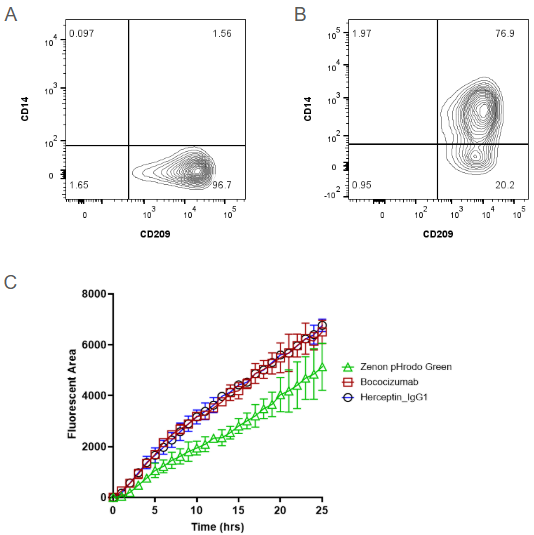


**Figure S2**. The fully differentiated immature DCs exhibit very low CD14 expression and high CD209 expression, as demonstrated in Figure A. In contrast, the poorly differentiated immature DCs typically show high CD14 expression and low CD209 expression, although they remain positive for CD209, as shown in Figure B. A representative DC internalization plot using Zenon pHrodo with poorly differentiated immature DCs is presented in Figure C. Notably, there is minimal separation between herceptin and bococizumab due to the increased uptake of herceptin.


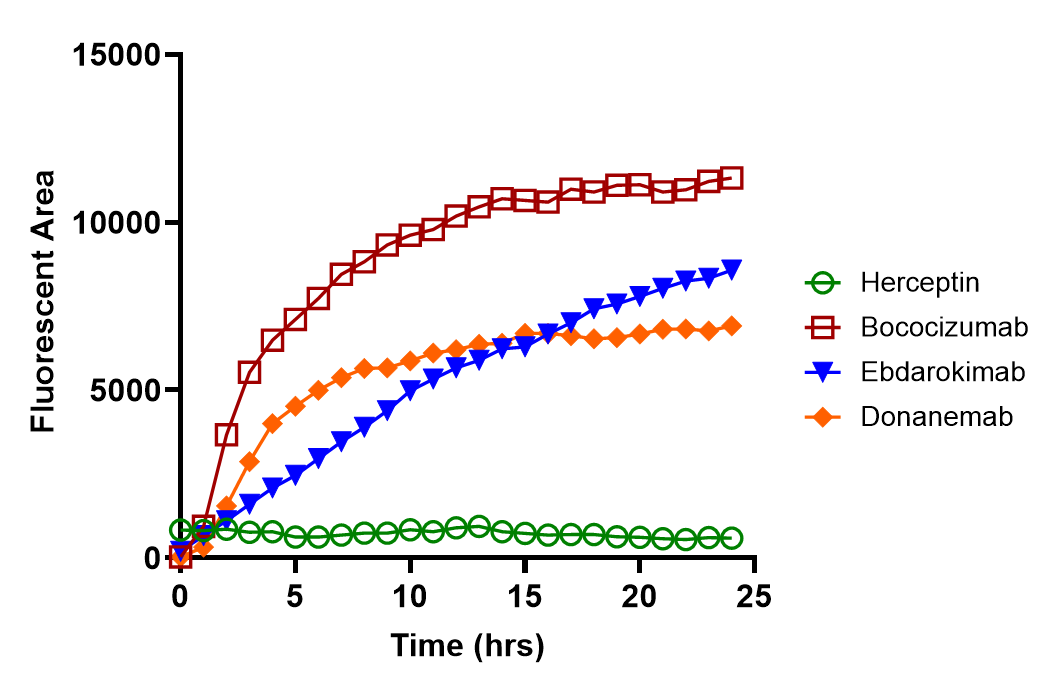


**Figure S3**. The internalization of BioTracker Orange labeled ebdarokimab crossed with that of donanemab at around 15 hr. Therefore, the timing of monitor internalization is important if only endpoint readout is used. Mean signal was shown from duplicate wells.


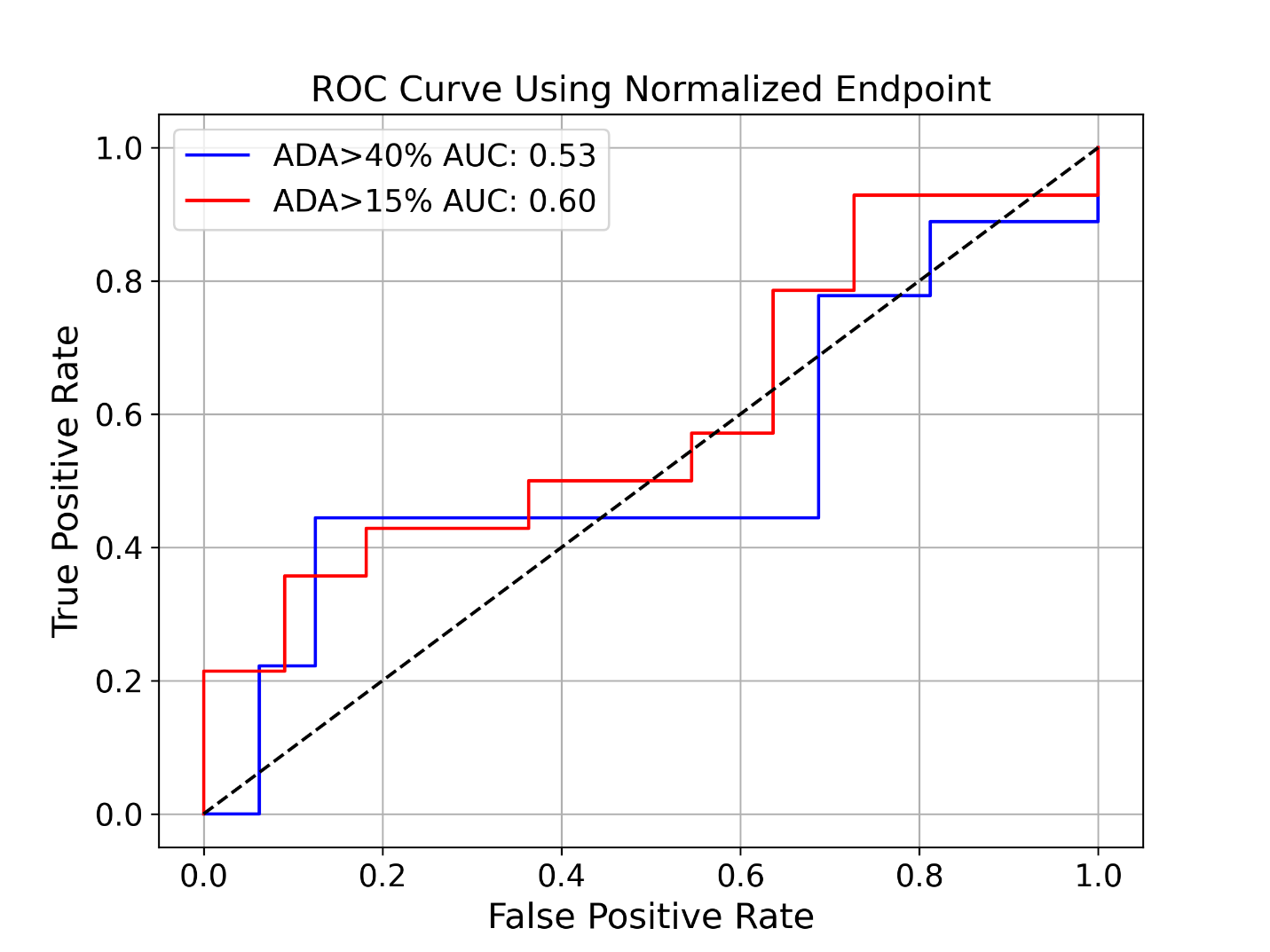


**Figure S4**. ROC curves from logistic regression modeling are presented using our previously developed flow cytometry-based internalization assay. The blue curve illustrates the AUC for ADA>40% versus ADA<40%, while the red curve illustrates the AUC for ADA > 15 versus ADA<15%.


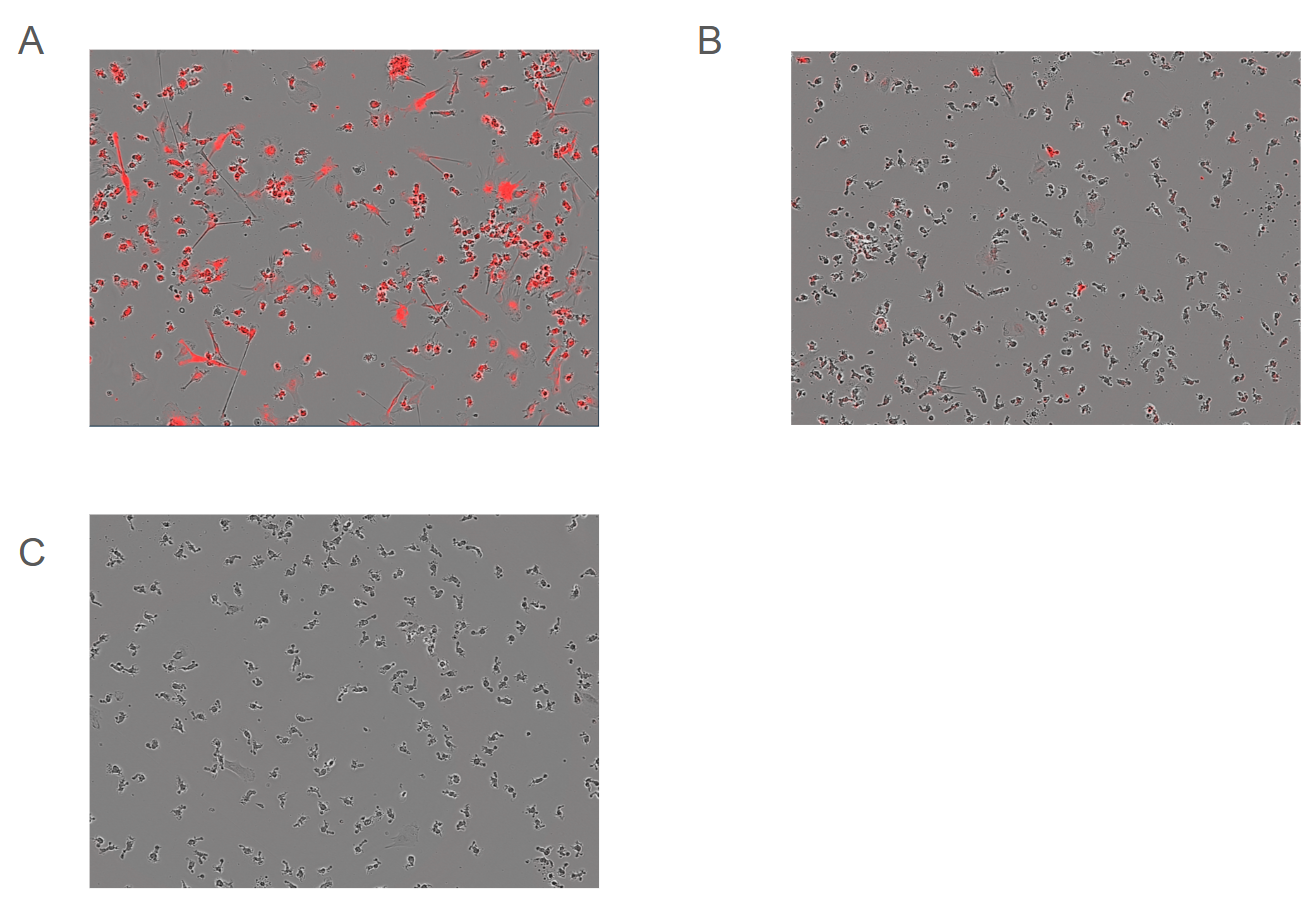


**Figure S5**. Images of DCs after 10-hr treatment with bococizumab (A), herceptin (B), and assay medium only (C). The DCs treated with bococizumab showed much more elongated dendrites and expanded cell area, which are typical characteristics of mature dendritic cells. This observation is consistent with the report that bococizumab is able to activate immature dendritic cells in vitro (1). However, a rigorous assessment of DC maturation would require a separate, comprehensive investigation using multi-parameter flow cytometry to quantify changes in specific cell surface markers (e.g., CD83, CD86, HLA-DR).


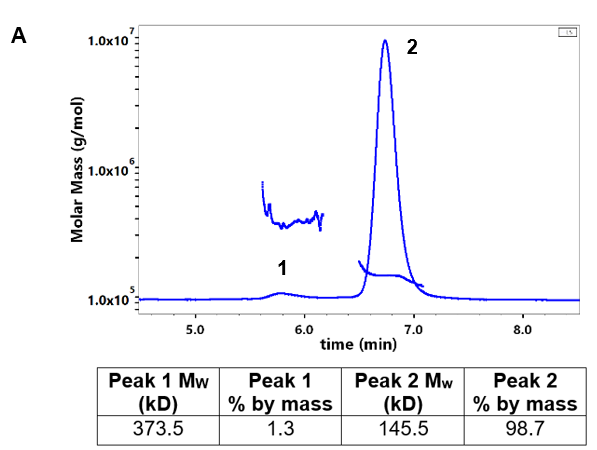


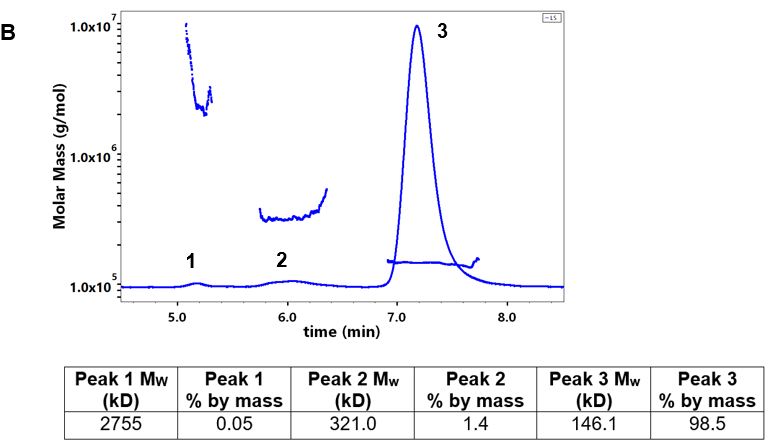


**Figure S6**. **Representative light scattering chromatograms for BioTracker Orange (BO) conjugates.** Peaks present in each sample are numbered in order of increasing the retention time. Percent by mass and molar mass (M_w_) for each peak are annotated in tables below each chromatogram. BO-donanemab (A) contains two species: A peak that is approximately dimer M_w_ (Peak 1) and monomer (Peak 2). However, three species are present in BO-daratumumab (B): HMWS (Peak 1), dimer (Peak 2), and monomer (Peak 3).


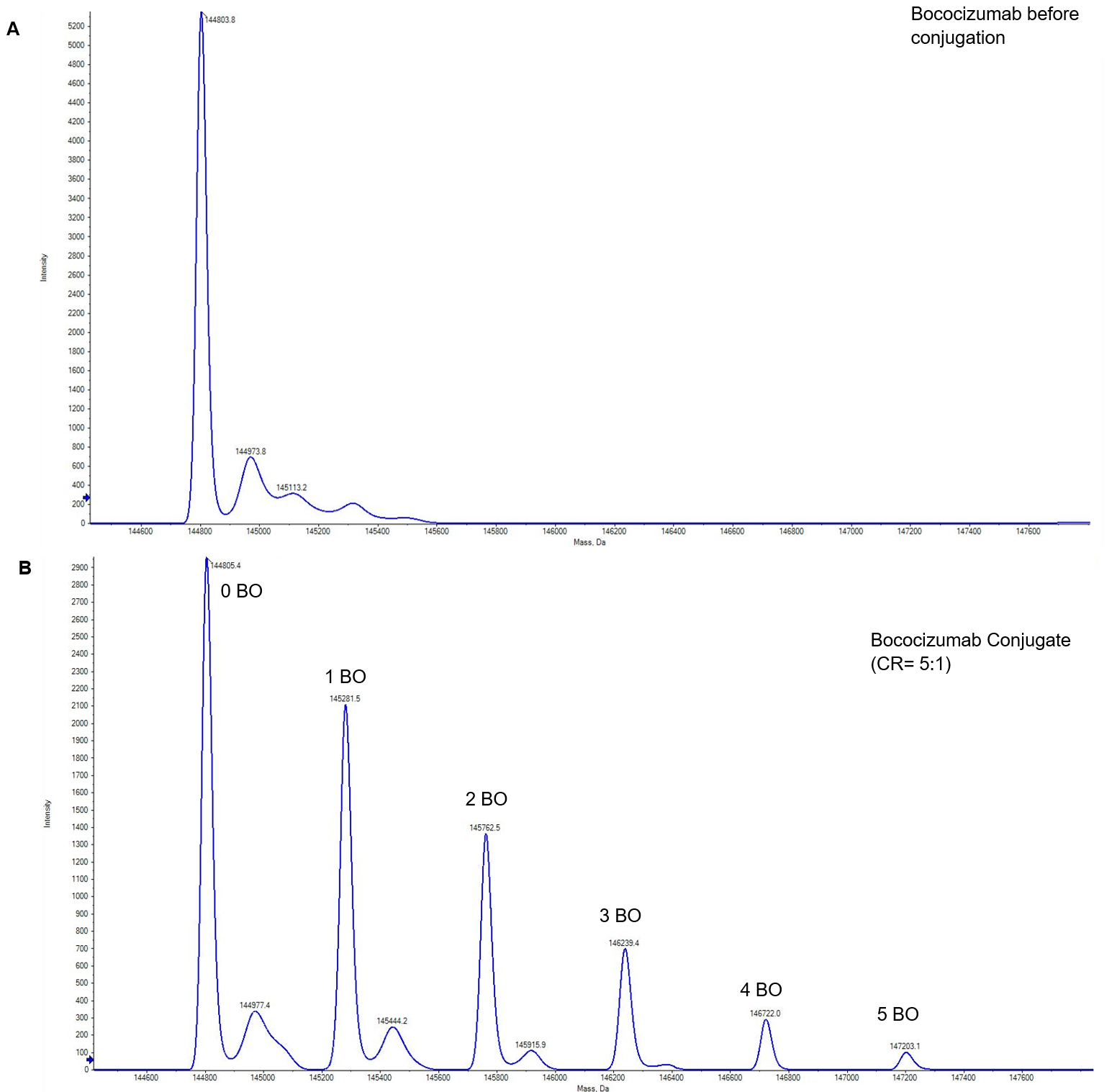


**Figure S7**. **Representative mass spectrum for BioTracker Orange (BO) conjugate.** (A) mass spectrum of bococizumab before conjugation; (B) mass spectrum of bococizumab after labeling with BO at a dye-to-protein challenge ratio (CR) of 5:1. The peak of each BO conjugate species is annotated.

1. Wickramarachchi D, Steeno G, You Z, Shaik S, Lepsy C, Xue L. Fit-for-Purpose Validation and Establishment of Assay Acceptance and Reporting Criteria of Dendritic Cell Activation Assay Contributing to the Assessment of Immunogenicity Risk. *Aaps j* (2020) 22(5):114. Epub 20200824. doi: 10.1208/s12248-020-00491-8.
