# Supplementary material for: A real-time imaging approach to quantify dendritic cell internalization for immunogenicity risk assessment of biotherapeutics
Source: Front Immunol. 2025 Sep 12;16:1632302. doi: 10.3389/fimmu.2025.1632302 (PMC12463596; doi:10.3389/fimmu.2025.1632302)
Supplement: Supplementary file 1 [file Presentation1.zip › Suppl. Tables.DOCX]

**Supplementary Tables**

**Establishing a Real-time Imaging Based Direct Labeling DC Internalization Assay to Inform the Immunogenicity Risk of Biotherapeutics**

Zhaojun Yin*, Peter Tran, Joyce Guerrero, Justin Low, Qing Xie, Kun Peng

Departments of Bioanalytical Sciences

CONTACT: Department of BioAnalytical Sciences, Genentech, Inc., South San Francisco, CA, USA.
*Corresponding author

**KEYWORDS**

Immunogenicity prediction, dendritic cell, IncuCyte^Ⓡ^, internalization assay, real-time imaging

**Table S1**. Challenge ratio (CR, dye:protein) and percentage of 0 - 10 Biotracker Orange (BO)/mAb determined by intact LC-MS for HuA33, golimumab, bococizumab and herceptin. ND: not detected.

| Drug | CR | 0 BO (%) | 1 BO (%) | 2 BO (%) | 3 BO (%) | 4 BO (%) | 5 BO (%) | 6 BO (%) | 7 BO (%) | 8 BO (%) | 9 BO (%) | 10 BO (%) |
| --- | --- | --- | --- | --- | --- | --- | --- | --- | --- | --- | --- | --- |
| HuA33 | 3:1 | 56.4 | 26.9 | 11.1 | 4.6 | 1.0 | ND | ND | ND | ND | ND | ND |
|  | 5:1 | 39.6 | 27.4 | 17.4 | 10.1 | 4.1 | 1.1 | 0.4 | ND | ND | ND | ND |
|  | 10:1 | 13.1 | 17.3 | 19.0 | 17.3 | 13.2 | 8.8 | 5.2 | 3.5 | 1.9 | 0.8 | ND |
| Golimumab | 3:1 | 48.1 | 30.4 | 14.2 | 6.0 | 1.3 | ND | ND | ND | ND | ND | ND |
|  | 5:1 | 31.3 | 28.0 | 20.1 | 12.4 | 5.7 | 1.7 | 0.7 | ND | ND | ND | ND |
|  | 10:1 | 11.2 | 17.6 | 20.0 | 18.6 | 14.1 | 9.2 | 4.9 | 2.6 | 1.3 | 0.5 | ND |
| Bococizumab | 3:1 | 54.6 | 31.6 | 10.9 | 2.8 | 0.2 | ND | ND | ND | ND | ND | ND |
|  | 5:1 | 41.3 | 30.9 | 17.2 | 8.1 | 2.5 | ND | ND | ND | ND | ND | ND |
|  | 10:1 | 17.0 | 21.4 | 22.2 | 17.6 | 11.8 | 6.0 | 2.6 | 1.0 | 0.4 | ND | ND |
| Herceptin | 3:1 | 51.4 | 29.4 | 13.2 | 5 | 0.9 | ND | ND | ND | ND | ND | ND |
|  | 5:1 | 34.8 | 27.2 | 18.9 | 11.5 | 5.4 | 1.8 | 0.5 | ND | ND | ND | ND |
|  | 10:1 | 11.3 | 16.0 | 18.2 | 17.4 | 14.3 | 10.3 | 6.3 | 3.7 | 1.6 | 0.8 | 0.2 |

**Table S2:** SEC-MALS-derived %monomer and %HMWS (high molecular weight species) for BioTracker Orange conjugates.

| **BO Conjugate** | **Challenge Ratio (dye:protein)** | **% Monomer** | **% HMWS > 1 MDa** |
| --- | --- | --- | --- |
| HuA33 | 3:1 | 99.3 | 0 |
| HuA33 | 5:1 | 99.7 | 0 |
| HuA33 | 10:1 | 99.3 | 0 |
| golimumab | 3:1 | 99.1 | 0 |
| golimumab | 5:1 | 99.4 | 0 |
| golimumab | 10:1 | 97.8 | 0 |
| bococizumab | 3:1 | 99.7 | 0 |
| bococizumab | 5:1 | 99.3 | 0 |
| bococizumab | 10:1 | 99.6 | 0 |
| Herceptin | 3:1 | 99.8 | 0 |
| Herceptin | 5:1 | 100 | 0 |
| Herceptin | 10:1 | 99.9 | 0.09 |
| briakinumab | 5:1 | 98.5 | 0 |
| tildrakizumab | 5:1 | 98.7 | 0.02 |
| ebdarokimab | 5:1 | 97.8 | 1.16 |
| satralizumab | 5:1 | 99.3 | 0.02 |
| sarilumab | 5:1 | 97.5 | 0.04 |
| ustekinumab | 5:1 | 99.6 | 0 |
| bimekizumab | 5:1 | 96.7 | 0.03 |
| elotuzumab | 5:1 | 98.4 | 0 |
| canakinumab | 5:1 | 99.4 | 0.02 |
| ixekizumab | 5:1 | 98.4 | 0.03 |
| secukinumab | 5:1 | 98.4 | 0 |
| eptinezumab | 5:1 | 98.3 | 0 |
| vedolizumab | 5:1 | 97.5 | 0 |
| lecanemab | 5:1 | 98.0 | 0 |
| donanemab | 5:1 | 98.7 | 0 |
| dezamizumab | 5:1 | 99.0 | 0 |
| adacanumab | 5:1 | 98.8 | 0 |
| daratumumab | 5:1 | 98.5 | 0.05 |
| tocilizumab | 5:1 | 99.2 | 0 |
